# Impact of hospital volume on failure to rescue for complications requiring reoperation after elective colorectal surgery: multicentre propensity score–matched cohort study

**DOI:** 10.1093/bjsopen/zrae025

**Published:** 2024-04-10

**Authors:** Marie T Grönroos-Korhonen, Laura E Koskenvuo, Panu J Mentula, Taina P Nykänen, Selja K Koskensalo, Ari K Leppäniemi, Ville J Sallinen

**Affiliations:** Gastroenterological Surgery, Helsinki University Hospital and University of Helsinki, Helsinki, Finland; Gastroenterological Surgery, Päijät-Häme Central Hospital, Lahti, Finland; Gastroenterological Surgery, Helsinki University Hospital and University of Helsinki, Helsinki, Finland; Gastroenterological Surgery, Helsinki University Hospital and University of Helsinki, Helsinki, Finland; Gastroenterological Surgery, Hyvinkää Hospital, Helsinki, Finland; Gastroenterological Surgery, Helsinki University Hospital and University of Helsinki, Helsinki, Finland; Gastroenterological Surgery, Helsinki University Hospital and University of Helsinki, Helsinki, Finland; Gastroenterological Surgery, Helsinki University Hospital and University of Helsinki, Helsinki, Finland; Transplantation and Liver Surgery, Helsinki University Hospital and University of Helsinki, Helsinki, Finland

## Abstract

**Background:**

It has previously been reported that there are similar reoperation rates after elective colorectal surgery but higher failure-to-rescue (FTR) rates in low-volume hospitals (LVHs) *versus* high-volume hospitals (HVHs). This study assessed the effect of hospital volume on reoperation rate and FTR after reoperation following elective colorectal surgery in a matched cohort.

**Methods:**

Population-based retrospective multicentre cohort study of adult patients undergoing reoperation for a complication after an elective, non-centralized colorectal operation between 2006 and 2017 in 11 hospitals. Hospitals were divided into either HVHs (3 hospitals, median ≥126 resections per year) or LVHs (8 hospitals, <126 resections per year). Patients were propensity score–matched (PSM) for baseline characteristics as well as indication and type of elective surgery. Primary outcome was FTR.

**Results:**

A total of 6428 and 3020 elective colorectal resections were carried out in HVHs and LVHs, of which 217 (3.4%) and 165 (5.5%) underwent reoperation (*P* < 0.001), respectively. After PSM, 142 patients undergoing reoperation remained in both HVH and LVH groups for final analyses. FTR rate was 7.7% in HVHs and 10.6% in LVHs (*P* = 0.410). The median Comprehensive Complication Index was 21.8 in HVHs and 29.6 in LVHs (*P* = 0.045). There was no difference in median ICU-free days, length of stay, the risk for permanent ostomy or overall survival between the groups.

**Conclusion:**

The reoperation rate and postoperative complication burden was higher in LVHs with no significant difference in FTR compared with HVHs.

## Teaser

This population-based retrospective multicentre cohort study compared results after reoperation for complications in colorectal surgery between high- and low-volume hospitals. We found the reoperation risk and cumulative burden of complications to be higher in low-volume hospitals, whereas failure to rescue remained similar.

## Introduction

Colorectal surgery is associated with a risk of 20–37% postoperative complications, 6–8% emergency reoperation rate and 2–19% risk of mortality^1–6^. Previous studies have demonstrated improved short- and long-term outcomes for high-volume hospitals (HVHs) in complex oncological resections such as pancreatectomy, liver resections, urology, oesophagectomy^7–11^ and in colorectal cancer surgery^12,13^. Studies looking at differences in short-term outcomes have reported that postoperative complications and reoperations occur at a similar rate in HVHs *versus* low-volume hospitals (LVHs), and even in high- *versus* low-income countries^14–17^. The difference in short-term outcomes between HVHs and LVHs has been thought to arise mainly from mortality rate after a complication has occurred that is failure-to-rescue (FTR), which has been reported to be lower in HVHs^15,18–21^. However, there are also contradictory reports^22,23^. All studies are registry-based retrospective studies, and although they have used multivariable models to adjust outcomes, none have utilized matching of patients using propensity score. In order to improve quality of care, the mechanisms by which the outcomes are mediated are of the utmost importance for the healthcare district administration making decisions on the distribution (or centralization) of care.

The aim of this study was to assess the effect of hospital volume on reoperation rate and FTR after emergency reoperation following elective colorectal surgery in a defined district in Southern Finland. To diminish the effect of patient selection bias, propensity score matching (PSM) was performed.

## Methods

Adult (over 18 years old) patients undergoing colorectal surgery between 1 January 2006 and 31 December 2017 in the Hospital District of Helsinki and Uusimaa (HUS) were screened. The HUS hospital district comprises 11 hospitals (3 university hospitals acting as both tertiary and secondary referral centres and 8 secondary referral centres) and serves a population of 1.7 million within a geographically defined area of 12,800 km^2^ in Southern Finland. All 11 hospitals are teaching hospitals supervising surgical residents. The hospitals work in close collaboration and patients may be transferred to another hospital for emergency operations if required, such as the need for specific surgical, anaesthetic or interventional radiological expertise. Patients were identified and the number of colorectal procedures performed annually in each hospital was obtained from the electronic patient records by interrogating the Nordic Medico-Statistical Committee (Surgical Procedural codes for elective colorectal resection (JFB20-JFB97, JFH00-JFH11, JFH96, JGB03-JGB11, JGB96-97)), which includes right- and left-sided hemicolectomies, subtotal colectomies and anterior rectal resections. Proctocolectomies and abdominoperineal resections were excluded because of centralization of these procedures. The first elective colorectal operation will be referred to as the index operation. Identified patients were assessed from the electronic records for a subsequent emergency reoperation within 30 days from the index operation, and these patients formed the final study cohort. Only operations that were due to a (suspected) complication and directly related to the index operation were considered as a reoperation. The patient records of these patients were assessed and data regarding pre-, peri- and postoperative characteristics were manually extracted. All hospitals used the same shared electronic patient record system during the study period.

Patients were divided into HVHs and LVHs depending on which hospital type the index elective operation was performed in. The cut-off used to define HVH and LVH in earlier reports has varied between five and 530 with a median of 126 operations per year^24^. In this study, 126 was hence used as the cut-off, yielding three hospitals classified as HVHs, also acting as university hospitals, and eight as LVHs.

The Charlson Comorbidity Index, which is a weighted index predicting 10-year survival based on patients’ co-morbidities, was calculated for all patients^25^. Primary outcome was FTR (defined as mortality for any cause within 90 days after a reoperation). Secondary outcomes included Comprehensive Complication Index (CCI) reflecting overall postoperative morbidity, length of stay (LOS)^26^, ICU-free days, permanent ostomy rate and overall survival.

FTR was defined as mortality for any cause within 90 days after a reoperation. CCI is the sum of all complications that are weighted on their severity based on the Clavien–Dindo classification (CCI=√(wC + wC2+….wCx)/2) and has values ranging from 0 to 100^27^. CCI was calculated for all patients within 30 days of the reoperation. CCI distribution was visualized with a boxplot. ICU-free days were defined as days alive within 30 days postoperative minus days spent in the ICU (range 0–30). LOS was defined as the time (days) between the (first) reoperation and the day of discharge. Permanent ostomy was defined as no stoma reversal within 2 years of reoperation, as nearly all reversals are performed within that time frame^28^. Overall survival was estimated using the Kaplan–Meier function from the reoperation until death for any reason and censored at the last day of follow-up.

PSM was carried out using variables clinically judged to impact patient selection and included age, BMI, sex, smoking, intake of oral corticosteroids, immunosuppression or anticoagulation, severe kidney disease, severe liver disease, congestive heart failure, ischaemic heart disease, chronic obstructive pulmonary disease, dementia, peripheral vascular disease, any cerebrovascular incident, diabetes mellitus, indication for surgery (diverticulosis, malignant or premalignant disease and inflammatory bowel disease) and type of resection (right- or left-sided, subtotal and rectal resection).

Match tolerance was defined as 0.2 s.d. of logit of the propensity score. Matching was done manually 1:1 using the ‘nearest neighbour’ method. For evaluating the effect of matching, the standardized mean difference (SMD) was defined before and after matching for pre- and intraoperative variables from the index operation. The groups were considered comparable if SMD was <0.10.

Categorical variables were compared using the chi-squared test, or Fisher’s exact test if expected cases in one cell were fewer than five. Normality of distribution of continuous variables was tested using the Kolmogorov–Smirnov test. As all continuous variables were non-normally distributed, they were analysed using the Mann–Whitney U test. Effect sizes for continuous non-normally distributed variables were calculated using *R* = *Z*/√(*N*), where <0.1 is a very small effect, 0.1–0.3 is a small effect, 0.3–0.5 is a medium effect and >0.5 is a large effect. Two-tailed *P* less than 0.05 was considered statistically significant. Statistical analyses were conducted using IBM SPSS software, version 27. Patients with missing values were excluded from analyses of that particular variable. The study is reported according to STROBE guidelines^29^.

Helsinki University Hospital Institutional Review Board approved the study. Ethical committee approval was not needed as this was a retrospective review of patient records.

## Results

After excluding already centralized procedures, 9429 patients underwent an elective colorectal resection during the study period. Of these operations, 6428 (68.2%) were performed in HVHs and 3020 (32.0%) in LVHs. The exact number of operations in each hospital is reported in *Table S1*. A total of 382 (4.1%) patients had a reoperation within 30 days and these patients formed the final study cohort. Of these patients, 217 (56.8%) were primarily operated in HVHs and 165 (43.2%) in LVHs. The reoperation rate was 3.4% in HVHs and 5.5% in LVHs (*P* < 0.001). This difference in reoperation rate means a surplus of 62 reoperations in LVHs during the study period of 12 years and, on average, an excess of 0.6 reoperations per year per hospital in LVHs if compared to the rate in HVHs. In 28 patients (7.3%) the first reoperation was done in a different hospital type than the index operation (25 patients were referred from an LVH to an HVH and 3 from an HVH to an LVH. All patients were stratified to HVH or LVH based on hospital of index elective operation regardless of where the reoperation was carried out). The reasons for transfer from LVH to HVH were the need for computed tomography (*n* = 1), need for ICU care (*n* = 3) and lack of operating room resource or lack of gastrointestinal surgeon capable of dealing with the complication (*n* = 21).

**Fig. 1:**
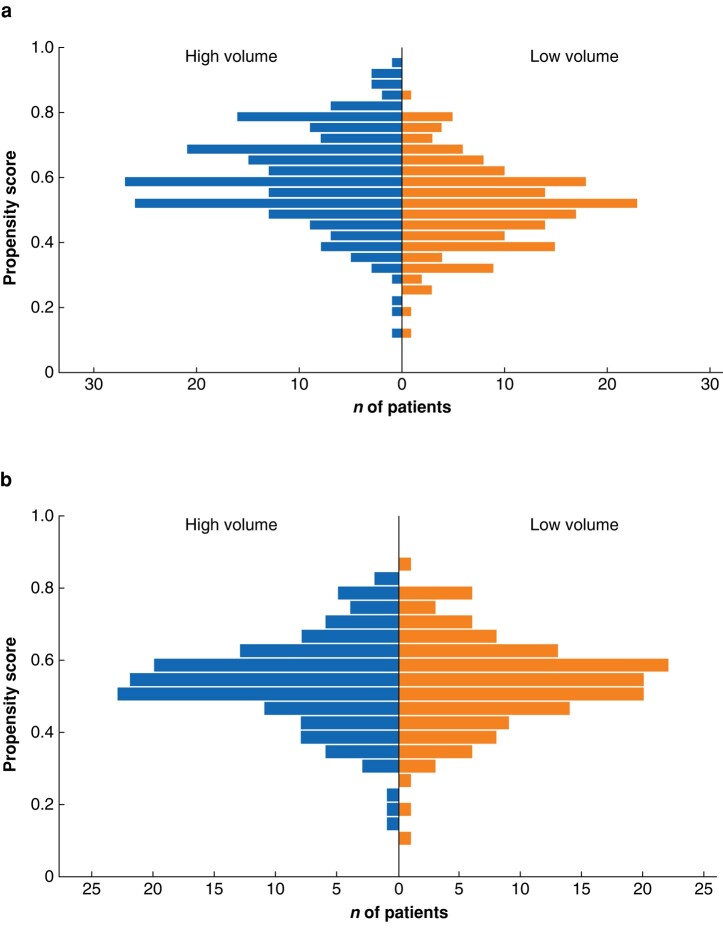
Mirrored histogram showing the propensity score distribution. **a** before matching, **b** after matching.

After PSM, 284 patients, 142 in both HVHs and LVHs, remained. Patients operated in HVHs had more co-morbidities than patients in LVHs before PSM (*Table 1*). There were no significant differences in indications or procedures performed in the index operation. Rectal resections were slightly more common in HVHs (12.1% *versus* 7.1%) and protective ostomies were more frequently done in HVHs (7.0% *versus* 3.6%) before PSM (*Table 1*). After matching, SMD improved significantly for most of the variables, making the two groups comparable. This is visualized with a mirrored histogram showing the propensity score distribution and overlapping in unmatched and matched samples (*Fig. 1*).

**Table 1:** Patient demographics, comorbidities and index operation details

	Original			Matched		
	High-volume hospital (*n* = 214) *n* (%) or median (i.q.r.)	Low-volume hospital (*n* = 168) *n* (%) or median (i.q.r.)	SMD before matching	High-volume hospital (*n* = 142) *n* (%) or median (i.q.r.)	Low-volume hospital (*n* = 142) *n* (%) or median (i.q.r.)	SMD after matching
**Sex**						
Male	138 (63.6)	109 (64.9)	0.200	87 (61.3)	90 (63.4)	0.043
Female	78 (36.4)	59 (35.1)		55 (38.8)	52 (36.7)	
Age (years)	68.4 (56.9–75.4)	65.1 (57.1–74.3)	0.052	67.2 (56.2–74.5)	64.7 (57.1–74.9)	0.021
BMI (kg/m^2^)	25.3 (22.4–29.0)	26.8 (23.8–29.8)	0.273	25.6 (23.0–30.0)	26.7 (23.7–29.7)	0.066
Current smoker	23 (10.7)	19 (11.3)	0.018	15 (10.6)	18 (12.7)	0.066
**Medication**						
Anticoagulation	40 (18.7)	23 (13.7)	0.135	19 (13.4)	22 (15.5)	0.060
Immunosuppressants	13 (6.1)	6 (3.6)	0.115	4 (2.8)	5 (3.5)	0.040
Corticosteroids	12 (5.6)	8 (4.8)	0.153	8 (5.6)	7 (4.9)	0.031
Previous abdominal operations	95 (44.4)	58 (34.5)	0.202	60 (42.3)	50 (35.2)	0.152
Charlson Comorbidity Index	4 (0–6)	4 (0–6)	0.048	3 (0–6)	4 (0–6)	0.056
Atrial fibrillation	31 (14.5)	18 (10.7)	0.113	20 (14.1)	18 (12.7)	0.041
Ischaemic heart disease	34 (1.4)	20 (11.9)	0.114	17 (12.0)	16 (11.3)	0.022
Myocardial infarct	21 (9.8)	9 (5.4)	0.168	10 (7.0)	5 (3.5)	0.157
Congestive heart failure	31 (14.5)	16 (9.5)	0.151	13 (9.2)	14 (5.6)	0.024
Peripheral vascular disease	14 (6.5)	5 (3.0)	0.164	5 (3.5)	5 (3.5)	0.000
Dementia	7 (3.3)	3 (1.8)	0.093	2 (1.4)	3 (2.1)	0.053
Cerebrovascular accident/transient ischaemic attack	17 (7.9)	9 (5.4)	0.103	6 (4.2)	8 (5.6)	0.065
Chronic obstructive pulmonary disease	18 (8.4)	6 (3.6)	0.200	5 (3.5)	6 (4.2)	0.036
Connective tissue disease	9 (4.2)	9 (5.4)	0.054	6 (4.2)	7 (4.9)	0.034
Diabetes mellitus	34 (15.9)	23 (13.7)	0.070	19 (13.4)	18 (12.7)	0.021
Peptic ulcer	6 (2.8)	3 (1.8)	0.067	4 (2.8)	3 (2.1)	0.045
Liver disease	7 (3.3)	2 (1.2)	0.137	4 (2.8)	2 (1.4)	0.098
Hemiplegia	11 (5.1)	3 (1.8)	0.179	3 (2.1)	2 (1.4)	0.053
Solid tumour, local	95 (44.4)	81 (48.2)	0.077	62 (43.7)	65 (45.8)	0.042
Solid tumour, metastatic	19 (8.9)	15 (8.9)	0.002	13 (9.2)	13 (9.2)	0.000
Leukaemia	2 (0.9)	1 (0.6)	0.038	1 (0.7)	1 (0.7)	0.000
Lymphoma	2 (0.9)	3 (1.9)	0.075	1 (0.7)	2 (1.4)	0.069
Acquired immune deficiency syndrome	0	0	–	0	0	
Chronic kidney disease	12 (5.6)	4 (2.4)	0.161	5 (3.5)	4 (2.8)	0.040
Previous thrombosis	16 (7.5)	6 (3.6)	0.168	8 (5.6)	5 (3.5)	0.101
**Surgical approach**						
Laparoscopy	112 (52.3)	65 (38.7)	0.275	79 (55.6)	58 (40.8)	0.298
Open	71 (33.2)	74 (44.1))	0.225	43 (30.3)	59 (41.5)	0.236
Converted	31 (14.5)	29 (17.3)	0.076	20 (14.1)	25 (17.6)	0.096
Operating time (min)*	140 (112–189)	154 (114.0–211.3)	0.002	138.0 (112.0–189.0)	154 (110–206)	0.004
Blood loss (ml)†	150 (50–400)	150 (75–350)	0.001	100 (50–400)	150 (50–350)	0.026
**Reason for surgery**						
Colorectal cancer or premalignant lesion	134 (62.6)	111 (66.1)	0.072	88 (62.0)	91 (64.1)	0.044
Diverticulosis	49 (22.9)	41 (24.4)	0.035	38 (26.8)	35 (24.6)	0.048
Benign large bowel obstruction	10 (4.7)	4 (2.4)	0.087	5 (3.5)	5(3.5)	0.000
Inflammatory bowel disease	13 (6.1)	6 (3.6)	0.115	5 (3.5)	6 (4.2)	0.036
Other malignancy	3 (1.4)	3 (1.8)	0.006	3 (2.1)	3 (2.1)	0.000
**Other resection ‡**	5 (2.3)	3 (1.8)	0.054	3 (2.1)	3 (2.1)	0.053
Right-sided colectomy	79 (36.9)	55 (32.7)	0.087	48 (36.6)	50 (35.2)	0.030
Left-sided colectomy	104 (48.6)	92 (54.8)	0.111	77 (54.2)	73 (51.4)	0.042
Rectal resection	26 (12.1)	12 (7.1)	0.125	9 (6.3)	10 (7.0)	0.028
Subtotal colectomy	10 (4.7)	12 (7.1)	0.106	8 (5.6)	9 (6.3)	0.030
Faecal contamination	15 (7.0)	12 (7.1)	0.009	9 (6.3)	8 (5.6)	0.026
Primary anastomosis	211 (98.6)	166 (98.8)	0.019	141 (99.3)	140 (98.6)	0.069
Protective diversion	15 (7.0)	6 (3.6)	0.133	5 (3.5)	5 (3.5)	0.000

*Seventeen patients were missing data for operating time (10 in low-volume hospitals (LVHs) and 7 in high-volume hospitals (HVHs)). †Forty-five patients were missing data for blood loss (19 in LVHs and 26 in HVHs). ‡Other: iatrogenic lesion, *n* = 1; trauma, *n* = 1; endometriosis, *n* = 1; rectum prolapse, *n* = 1; hidradenitis, *n* = 1; polyposis, *n* = 2; other infection, *n* = 1; other benign lesion, *n* = 1. SMD, standardized mean difference.

The only medical history or index operation factors that did not meet the criteria of being comparable after matching (that is SMD < 0.1) were previous operations, myocardial infarction, previous thrombosis and operative technique (laparoscopy or laparotomy) (*Table 1*).

Details of reoperation are shown in *Table 2*. Intra-abdominal infection was the most common finding in reoperation in both HVHs (65.5%) and LVHs (62.0%), followed by intra-abdominal bleeding (20.4% in HVHs and 14.8% in LVHs) and fascial rupture (13.4% in LVHs and 11.3% in HVHs). Superficial wound infection was more common in LVHs than HVHs, 4.9% *versus* 0% (*P* = 0.007). There was no significant difference regarding surgical approach, blood loss or operating time. Procedures done in the reoperation were also similar for both LVHs and HVHs. Only resuturing of the anastomosis was performed more often in HVHs, 28 (19.7%) compared to 16 (11.3%) in LVHs. While time from index operation to reoperation was similar in HVHs and LVHs, more reoperations were carried out at night (between 22.00 and 8.00) in HVHs (*Table 2*). Although the distribution of different complications at reoperation was similar between HVHs and LVHs (shown in *Table 2*), the overall rate (complications per number of index elective operations) of intra-abdominal infection (93/6248 (1.4%) *versus* 88/3020 (2.9%), *P* < 0.001), bowel obstruction (8/6248 (0.1%) *versus* 17/3020 (0.6%), *P* = 0.001), wound infection (0/6248 (0%) *versus* 7/3020 (0.2%), *P* < 0.001) and fascial rupture (16/6248 (0.2%) *versus* 19/3020 (0.6%), *P* = 0.005) needing reoperation after elective colorectal surgery was higher in LVHs compared to HVHs. The rate of intra-abdominal bleeding (29/6248 (0.5%) *versus* 21/3020 (0.7%), *P* = 0.129) was similar.

**Table 2:** Reoperation details in propensity score–matched cohorts

	High-volume hospital (*n* = 142) *n* (%) or median (i.q.r.)	Low-volume hospital (*n* = 142) *n* (%) or median (i.q.r.)	*P*
Findings in reoperation*			
Intra-abdominal infection	93 (65.5)	88 (62.0)	0.537
Intra-abdominal bleeding	29 (20.4)	21 (14.8)	0.213
Fascial rupture	16 (11.3)	19 (13.4)	0.588
Bowel obstruction	8 (5.6)	17 (12.0)	0.059
Wound infection (superficial)	0	7 (4.9)	**0**.**007**
No findings (negative relaparotomy)	4 (2.8)	3 (2.1)	0.211
**Surgical approach**			
Open	127 (89.4)	121 (85.2)	0.285
Laparoscopic	5 (3.5)	6 (4.2)	0.758
Conversion	9 (6.4)	11 (7.7)	0.643
Operating time (min)†	91.0 (61.8–122.3)	90.0 (58.0–128.0)	0.912
Blood loss (ml)‡	150 (10.0–300.0)	100 (0.0–300.0)	0.300
Time to reoperation (days from index operation)	5.2 (3.2–8.6)	5.8 (3.2–9.2)	0.425
**Time of day of reoperation**			
8–16 h	34 (23.9)	35 (24.6)	0.890
16–22 h	55 (38.7)	64 (45.1)	0.279
22–6 h	50 (35.2)	34 (23.9)	**0**.**037**
**Procedures**			
Diversion			
Protective diversion	33 (23.2)	30 (21.1)	0.645
End ileostomy	2 (1.4)	7 (4.9)	0.092
End colostomy	27 (19.0)	27 (19.0)	0.977
Anastomotic stoma	1 (0.7)	2 (1.4)	0.566
Resection of anastomosis	53 (37.3)	52 (36.6)	0.866
New anastomosis	29 (20.4)	25 (17.6)	0.526
Resuturing of anastomosis	28 (19.7)	16 (11.3)	**0**.**046**
Fascial closure	15 (10.6)	20 (14.1)	0.367
Bowel resection (other than anastomosis)	12 (8.5)	15 (10.6)	0.544
Haemostasis and/or evacuation of haematoma*	14 (9.9)	9 (6.3)	0.269
Lysis of adhesions	6 (4.2)	11 (7.7)	0.211

*Sum of percentages is over 100% as one patient may have several findings simultaneously. Bold values indicate significance (*P* < 0.05). †Thirteen patients were missing data for operating time (9 in LVHs and 4 in HVHs). ‡Forty-two patients were missing data for blood loss (24 in LVHs and 18 in HVHs).

**Fig. 2:**
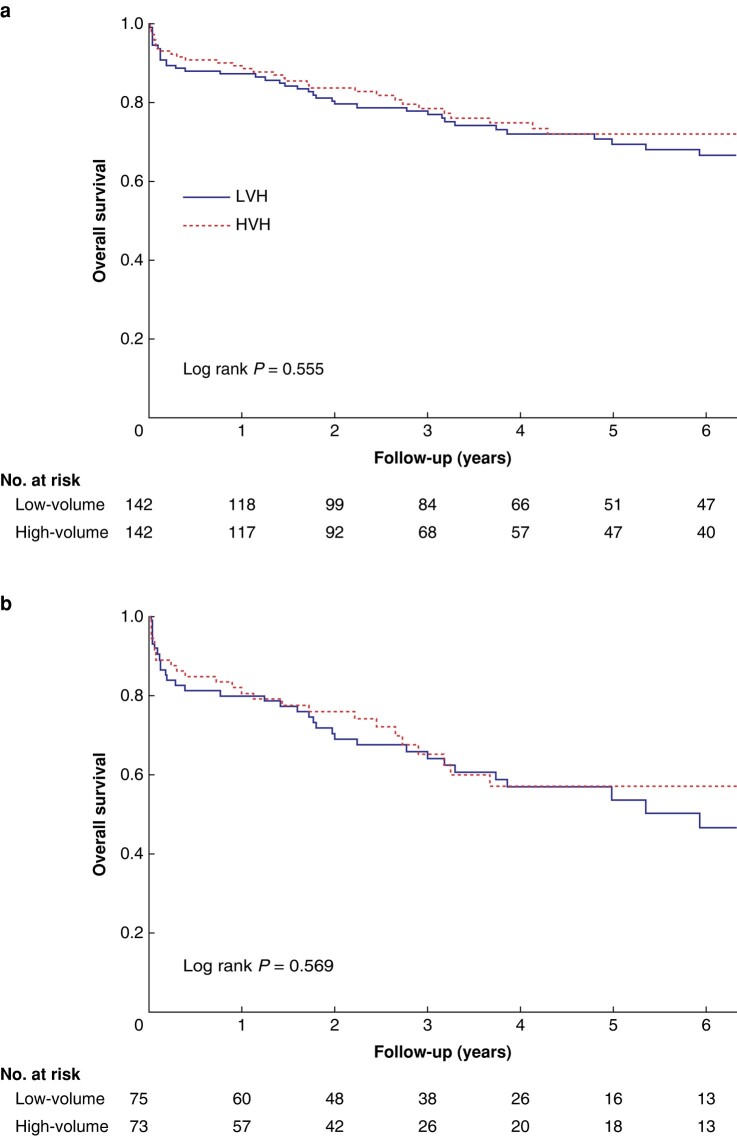
Survival after reoperation for colorectal surgery complication for (a) all patients and (b) patients with cancer as indication for index operation.

After PSM, FTR was 7.7% in HVHs and 10.6% in LVHs (*P* = 0.410; *Table 3*). Overall cumulative postoperative complications, assessed by CCI, were higher in LVHs (*Table 3*, *Fig. S1*) (median CCI 29.6 *versus* 21.8, *P* = 0.045). ICU-free days, length of hospital stay, and rate of permanent ostomy were similar for both groups (*Table 3*). The risk of permanent ostomy after anastomotic dehiscence was 14 of 82 patients (17.1%) for HVHs and 13 of 72 patients (18.1%) for LVHs. There was no difference in overall survival of all patients or patients with colorectal cancer within 10 years between HVHs and LVHs (*Fig. 2*). The median follow-up time was 40 (i.q.r. 19–82) months. Primary and secondary outcomes before matching are shown in *Table S2*.

**Table 3:** Primary and secondary outcomes after reoperation for a complication after elective colorectal surgery in propensity score–matched cohorts

	High-volume hospital (*n* = 142) *n* (%) or median (i.q.r.)	Low-volume hospital (*n* = 142) *n* (%) or median (i.q.r.)	OR (95% c.i.) or effect size (*r*)	*P*
FTR	11 (7.7)	15 (10.6)	0.711 (0.315–1.607)	0.410
CCI	21.8 (0.0–46.2)	29.6 (8.7–54.1)	0.119	0.045
ICU-free days*	30.0 (27.0–30.0)	30.0 (25.0–30.0)	0.080	0.179
Length of stay (days)	10.0 (6.0–17.0)	12 (6.0–18.0)	0.046	0.437
Permanent stoma	18 (12.7)	20 (14.1)	0.885 (0.447–1.755)	0.727

*Days alive within 30 days postoperative minus all days spent in the ICU (range 0–30). Effect size for continuous non-normally distributed variables was calculated using *r* = *Z*/√(*N*), where <0.1 is a very small effect, 0.1–0.3 is a small effect, 0.3–0.5 is a medium effect and >0.5 is a large effect. Bold values indicate significance (*P* < 0.05). CCI, Comprehensive Complication Index; FTR, failure to rescue.

## Discussion

In this population-based, propensity score–matched study, the reoperation rate after elective colorectal surgery was higher in LVHs compared with HVHs (3.4% *versus* 5.5%). This indicates approximately less than one extra reoperation per LVH per year, compared to HVHs. Although the FTR was statistically similar between HVHs and LVHs (8% *versus* 11%, respectively), a larger burden on cumulative complications after the reoperation was noted in LVHs (median CCI score 21.8 *versus* 29.6). The difference in the median scores (approximately 8 points) can be considered clinically significant as a 10-point difference in the CCI score reflects 1-grade difference in the Clavien–Dindo^30^. There was no difference in LOS, ICU-free days, frequency of permanent ostomy or long-term survival between the groups.

Reported reoperation rates after elective colorectal resection vary from 4.8% to 12.8%^31–33^. The reoperation rate for HVHs in this study was 3.4%, which could, therefore, be considered low, whereas the reoperation risk for LVHs was 5.5%, which is similar to previous reports. Similarly, the FTR rate in this study can be considered low in HVHs (8%) and average in LVHs (11%) compared to the literature reporting FTR for only elective operations between 7.9% and 13.2%^34^. A large Dutch study reported a variance of 0–29% in the FTR for all postoperative complications (not just reoperations), depending on hospital status. This suggests that HVHs are better at managing complications even though they seem to occur at similar rates regardless of hospital volume^15,21,35^. The authors' results do not support these results, as there was a significantly higher reoperation rate in LVHs. Of patients needing reoperation in LVHs, 16% were transferred to HVHs for reoperation, which might have decreased the FTR rates of LVHs and could explain the difference with earlier reports of higher FTR in LVHs. The exact reasons for the higher reoperation rate in LVHs remain unclear. No single complication type could be found to contribute more than another and the rate of different types of complications (infection, fascial rupture, bowel obstruction) were increased in LVHs. The incidence of negative relaparotomy was similar for HVHs and LVHs. Even though the FTR rate was unaffected, the increased postoperative morbidity among LVHs remains a concern. The reasons for increased morbidity can only be speculated. While the median time from index operation to reoperation was similar in both HVHs and LVHs, reoperations occurred more frequently during the night (between 22.00 and 8.00) in HVHs. This might reflect the better night-time resources in HVHs, which could have contributed to the lower postoperative morbidity in HVHs. These results support some of the previous reports showing reduced overall morbidity in favour of HVHs^12^.

Many reports show an inverse correlation between LOS and hospital volume for all patients after colorectal surgery^36,37^. No clear correlation was found in our study. Median LOS for colorectal surgery in general, including also non-complicated cases, varies between five and 14^38–40^. However, the results are not comparable with this study, because this cohort only comprises patients with at least CD 3b complications (that is, needing a reoperation) and LOS is expected to be higher after colorectal surgery in general (including patients who do not need reoperation). The risk of permanent ostomy was similar for HVHs and LVHs (13–14%). Relaparotomy increases the risk of open abdomen and stomas especially after anastomotic leak with high rates (27–57%) of permanent stoma^41^. The rate of permanent stoma in this study can be considered low, but this cohort also included patients without anastomotic dehiscence.

This study has certain limitations. This is a retrospective study with an inherent risk of bias, such as patient selection bias and potential imbalance of prognostic factors. Although PSM was used to mitigate the bias, PSM does not balance the unmeasured and unknown confounders. However, this study was not registry-based like many other similar studies, and all patients’ records were screened and data extracted manually, which improves data quality. The study cohort included all patients undergoing elective colorectal resection for various indications, which can be considered both a limitation and a strength. Interpreting mixed data is difficult, increasing the risk for bias. However, by only focusing on cancer patients, 55% of patients undergoing colorectal surgery would have been left out of the analyses. A major strength of this study is the population-based approach including all patients and public hospitals within a defined geographical area.

The implications of the study are multidimensional. The reoperation rate was higher in LVHs compared to HVHs. In contrast, the FTR rate was similar, which suggests that LVHs have capabilities (including the option of patient transfer to HVHs) to deal with colorectal complications that require a reoperation. This study does not support further centralization within this national population even though the reoperation rate was lower in HVHs. Removing elective colorectal surgery from LVHs could have negative consequences for the healthcare service in the area, so other options in improving care should be sought. These could include closer collaboration, standardized perioperative care and joint multidisciplinary teamwork between LVHs and HVHs. The colorectal surgeon teams in a hospital should be large enough to support specialization and out-of-hours availability. Of note, the earlier studies’ findings of equal reoperation rate but increased FTR in LVHs^14,18,19^ was not supported by these data. This implies that the driving mechanisms behind differences in outcomes between LVHs and HVHs are not universal and likely differ from country to country. This reinforces the need for the national healthcare system to have knowledge regarding the outcomes of HVHs *versus* LVHs in order to improve the overall care for all patients.

## Supplementary Material

zrae025_Supplementary_Data

## Data Availability

Study permissions do not allow sharing of individual patient data.
