# Supplementary material for: Impact of hospital volume on failure to rescue for complications requiring reoperation after elective colorectal surgery: multicentre propensity score–matched cohort study
Source: BJS Open. 2024 Apr 10;8(2):zrae025. doi: 10.1093/bjsopen/zrae025 (PMC11004787; doi:10.1093/bjsopen/zrae025)

THE IMPACT OF HOSPITAL VOLUME ON FAILURE TO RESCUE FOR COMPLICATIONS REQUIRING REOPERATION AFTER ELECTIVE COLORECTAL SURGERY: MULTICENTRE PROPENSITY SCORE MATCHED COHORT STUDY

Marie T Grönroos-Korhonen^1,4^ MD, Laura E Koskenvuo^1^ MD PhD, Panu J Mentula^1^ MD PhD, Taina P Nykänen^3^ MD PhD, Selja K Koskensalo^1^ MD PhD, Ari K Leppäniemi^1^ MD PhD, Ville J Sallinen^1,2^ MD PhD.

^1^Gastroenterological Surgery, Helsinki University Hospital and University of Helsinki, Helsinki, Finland

^2^Transplantation and Liver Surgery, Helsinki University Hospital and University of Helsinki, Helsinki, Finland

^3^Gastroenterological Surgery, Hyvinkää Hospital, Helsinki, Finland

^4^Gastroenterological Surgery, Päijät-Häme Central Hospital, Lahti, Finland

**Corresponding author.**

Ville Sallinen

Gastroenterological Surgery / Transplantation and Liver Surgery

Helsinki University Hospital and University of Helsinki

Haartmaninkatu 4

00029 Helsinki, Finland

Tel: +358-9-4711

Email: [ville.sallinen@helsinki.fi](mailto:ville.sallinen@helsinki.fi)

[**https://orcid.org/0000-0001-5394-4169**](https://orcid.org/0000-0001-5394-4169)

[**https://x.com/villesallinen?s=21&t=UPXxsHUNxGMx8q_ZdsQ8JQ**](https://x.com/villesallinen?s=21&t=UPXxsHUNxGMx8q_ZdsQ8JQ)

**Supplementary Materials - Index**

|  |  |
| --- | --- |
| **Supplementary Figures and Tables** |  |
| Supplementary table 1. Number of colorectal operations per hospital during the study period 2006-2017. | *page 3* |
| Supplementary table 2. Primary and secondary outcomes after reoperation for a complication after elective colorectal surgery before PSM. | *page 4* |
| Supplementary figure 1. Boxplot showing Comprehensive Complication Index distribution in patient undergoing reoperation after elective colorectal surgery in low versus high volume hospitals (median 29.6 versus 21.8, p=0.045). | *Page 5* |
|  |  |
|  |  |
|  |  |
|  |  |
|  |  |
|  |  |
|  |  |
|  |  |
|  |  |
|  |  |

**Supplementary table 1.** Number of colorectal operations per hospital during the study period 2006-2017.

| High-volume hospitals: | Number of elective colorectal operations after exclusion of centralised operations: | Mean per year | Number of elective colorectal operations: | Mean per year | Total number of colorectal operations: | Mean per year |
| --- | --- | --- | --- | --- | --- | --- |
| 1 | 2622 | 218.5 | 3164 | 263.7 | 3212 | 267.7 |
| 2 | 2529 | 210.8 | 1874 | 156.2 | 2525 | 210.4 |
| 3 | 1277 | 106.4 | 1887 | 157.3 | 3961 | 330.1 |
| Low-volume hospitals: |  |  |  |  |  |  |
| 4 | 851 | 70.9 | 901 | 75.1 | 1204 | 100.3 |
| 5 | 747 | 62.3 | 791 | 65.9 | 1055 | 87.9 |
| 6 | 389 | 32.4 | 397 | 33.1 | 587 | 48.9 |
| 7 | 380 | 31.7 | 390 | 32.5 | 523 | 43.6 |
| 8 | 285 | 23.8 | 285 | 23.8 | 285 | 23.8 |
| 9 | 135 | 11.3 | 136 | 11.3 | 140 | 11.7 |
| 10 | 140 | 11.7 | 140 | 11.7 | 158 | 13.2 |
| 11 | 94 | 7.8 | 95 | 7.9 | 101 | 8.3 |
|  |  |  |  |  |  |  |
| Mean | 859.0 | 62.6 | 914.5 | 76.2 | 1250.1 | 104.2 |

**Supplementary table 2.** Primary and secondary outcomes after reoperation for a complication after elective colorectal surgery before PSM.

|  | Before matching |  |  |  |
| --- | --- | --- | --- | --- |
|  | High volume hospital (n=217)  n (%) or median (IQR) | Low volume hospital (n=165)  n (%) or median (IQR) | Odds ratio (95% confidence interval) or effect size (r) | p-value |
| FTR | 19 (8.8) | 15 (9.1) | 0.960 (0.472-1.951) | 0.909 |
| Cci | 21.8 (0.0-46.2) | 27.6 (8.7-49.9) | 0.106 | **0.038** |
| ICU free days* | 30 (27.0-30.0) | 30 (24.0-30.0) | 0.095 | 0.0620 |
| Length of stay (days) | 10 (6.0-19.0) | 12 (6.0-18.0) | 0.061 | 0.236 |
| Permanent ostomy | 36 (16.6) | 24 (14.5) | 1.269 (0.721-2.233) | 0.408 |

*Days alive within 30 days postoperative minus all days spent in the ICU (range 0-30. Effect size for continuous non normally distributed variables were calculated using r = Z /√(N), where <0.1 is very small effect, 0.1 to 0.3 is small effect, 0.3 to 0.5 is medium effect and >0.5 is large effect. FTR - Failure to rescue. CCI - Comprehensive Complication Index.

**Supplementary figure 1.** Boxplot showing Comprehensive Complication Index distribution in patient undergoing reoperation after elective colorectal surgery in low versus high volume hospitals (median 29.6 versus 21.8, p=0.045).


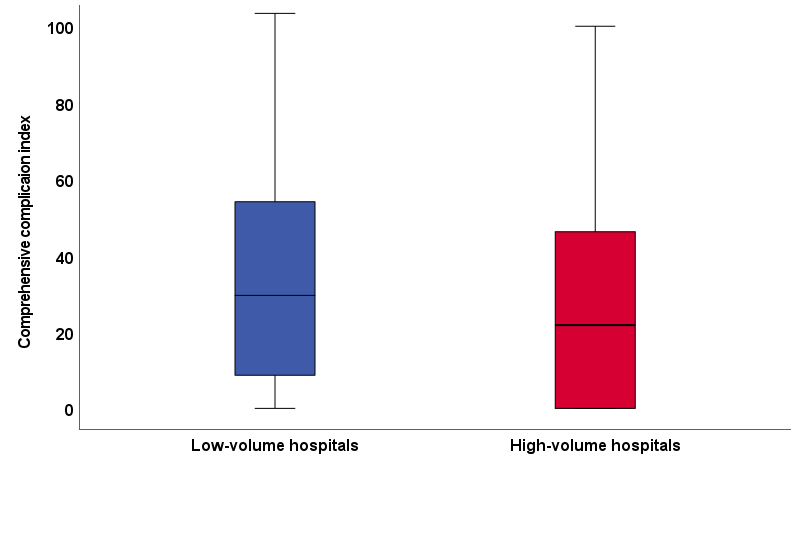

Supplement: zrae025_Supplementary_Data [file zrae025_supplementary_data.zip › Supplementary-Materials.docx]
